# A Comparison of the Accuracy of Four Age Estimation Methods Based on Panoramic Radiography of Developing Teeth

**DOI:** 10.15171/joddd.2015.015

**Published:** 2015-06-10

**Authors:** Shahrzad Javadinejad, Hajar Sekhavati, Roshanak Ghafari

**Affiliations:** ^1^Assistant Professor, Department of Pediatric Dentistry, Islamic Azad University Branch of Khorasgan, Iran; ^2^Post-graduate Student, Department of Pediatric Dentistry, Islamic Azad University Branch of Khorasgan, Iran; ^3^Department of Oral and Maxillofacial Radiology, Islamic Azad University Branch of Khorasgan, Iran

**Keywords:** Dental age determination, Forensic dentistry, panoramic radiography

## Abstract

***Background and aims.*** Tooth development is widely used in determining age and state of maturity. Dental age is of high importance in forensic and pediatric dentistry and also orthodontic treatment planning .The aim of this study was to compare the accuracy of four radiographic age estimation methods.

***Materials and methods.*** Orthopantomographic images of 537 healthy children (age: 3.9-14.5 years old) were evaluated. Dental age of the subjects was determined through Demirjian’s, Willem’s, Cameriere’s, and Smith’s methods. Differences and correlations between chronological and dental ages were assessed by paired t-tests and Pearson’s correlation analysis, respectively.

***Results.*** The mean chronological age of the subjects was 8.93 ± 2.04 years. Overestimations of age were observed following the use of Demirjian’s method (0.87 ± 1.00 years), Willem’s method (0.36 ± 0.87 years), and Smith’s method (0.06 ± 0.63 years). However, Cameriere’s method underestimated age by 0.19 ± 0.86 years. While paired t-tests revealed significant differences between the mean chronological age and ages determined by Demirjian’s, Willem’s, and Cameriere’s methods (P < 0.001), such a significant difference was absent between chronological age and dental age based on Smith’s method (P = 0.079). Pearson’s correlation analysis suggested linear correlations between chronological age and dental age determined by all four methods.

***Conclusion.*** Our findings indicated Smith’s method to have the highest accuracy among the four assessed methods. How-ever, all four methods can be used with acceptable accuracy.

## Introduction

Due to illegal immigrations and the growing incidence of natural disasters, age determination has gained increasing importance in legal medicine. Age also plays a critical role in pediatric dentistry, orthodontic treatment planning, and surgeries.^1^ A person’s physiological age is assessed based on his/her somatic maturation, i.e. maturation of functional body systems such as bones and teeth.^2^

Teeth undergo various development stages in the first 25 years of a human’s life and demonstrate secondary changes in the later years. On the other hand, they are not highly influenced by nutritional and endocrine factors. Hence, legal dentistry has turned into a dynamic and active field of medicine during the past two decades.^3^Numerous techniques have been suggested to determine age according to dental characteristics. Despite the use of time of tooth eruption in age determination, this index is widely affected by environmental factors including dental arch space, early extraction of primary teeth, tooth impaction, and tipping. Therefore, a number of approaches to age determination, e.g. evaluation of radiographic images,^4,5^ dental structure,^6-8^ Gustafson’s method,^9^ Lamendin’s method,^10,11^ and aspartic acid racemization,^12,13^ use tooth development stages as a more logical factor. Among the many advanced imaging technologies and radiographic images utilized to estimate age, viz. panoramic, periapical, cephalometric, and lateral oblique radiographs, panoramic radiographs are an accessible and inexpensive method to provide an outline of a person’s dental system maturity.^14^

Demirjian’s method is an extensively applied technique which utilizes radiographs and estimates age based on development stages of seven left mandibular permanent teeth.^15^ Willem’s method uses the same seven teeth and the eight development stages defined by Demirjian separately for boys and girls and calculates age by considering the set of indices for each tooth.^16^On the other hand, Cameriere’s method determines chronological age based on the relationship between age and measurement of open apices in tooth roots.^17^ Smith modified the technique developed by Moorrees et al^18^ and used 14 development stages for eight left mandibular teeth to estimate children’s age.^19^Since few studies have evaluated various age determination techniques among Iranian children, the present study compared the accuracy of Demirjian’s, Cameriere’s, Smith’s, and Willem’s methods in estimating the age of the mentioned population.

## Materials and Methods

This double blind study evaluated panoramic radiographs of 577 children (284 boys and 293 girls) in Isfahan, Iran. The radiographs, which had been ordered by dentists for diagnostic purposes, were taken in one of the oral and maxillofacial radiology centers in the city. Before the radiography, the children’s gender and birth date were recorded in a questionnaire. A radiologist trained the observer to use the related software and assess the radiographs. The inclusion criteria were age 3-15 years, absence of systemic diseases, dental anomalies, nutritional and endocrine problems, premature birth, and birth defect, and clear birth date and date of radiography. The exclusion criteria were too much magnification or minification, patient head rotation (unequal tooth size on the two sides), lack of one or more left mandibular permanent teeth, and low-quality radiographs.

All direct digital panoramic radiographs were obtained utilizing a Cranex D system (Soredex, Finland) via a charge-coupled device (CCD) and saved as .JPEG files. Analysis of the images in Romexis Viewer was then performed by a trained observer and under the supervision of an oral and maxillofacial radiologist. In order to measure intra-examiner reproducibility, 50 samples were reexamined at a two-week interval and the kappa score was calculated as 0.96.

The children’s chronological age (computed by subtracting their birth date from the radiography date) was compared with ages estimated by Demirjian’s, Cameriere’s, Smith’s, and Willem’s methods. Normality of data was assessed with Kolmogorov-Smirnov test. Pearson’s correlation analysis and paired t-tests were used to analyze the data in SPSS for Windows 10.0 (SPSS Inc., Chicago, IL, USA).

## Results

From the 577 children in the initial sample, one boy and 17 other children (seven boys and 10 girls) were excluded due to the congenital absence of mandibular lateral incisor and mandibular second premolar, respectively. Moreover, 12 boys and 10 girls were excluded because of various reasons such as low-quality images or extraction of a permanent tooth. Finally, 537 children and adolescents (264 boys and 273 girls) were studied.

Since Kolmogorov-Smirnov test results were not significant at P ≤ 0.05, scores of variables had normal distribution. The mean chronological ages of the whole sample, the girls, and the boys were 8.93, 8.95, and 8.90 years, respectively. Comparisons between the mean ages calculated by the four studied methods and the mean chronological age showed that while Demirjian’s, Willem’s, and Smith’s methods overestimated the children’s age, Cameriere’s method underestimated all values (Table 1).

**Table 1:** The mean age of all participants (n = 537), girls (n = 273), and boys (n = 264) calculated by different methods

**Method**		**Mean age (years)**	**Mean estimated age - mean chronological age (years)**	**Standard deviation**	**Minimum age (years)**	**Maximum age (years)**
**Chronological age**	**All**	8.93	-	2.04	3.90	14.50
	**Boys**	8.95	-	2.07		
	**Girls**	8.90	-	2.01		
**Demirjian’s method**	**All**	9.80	0.87	2.29	5.90	17.00
	**Boys**	9.86	0.90	2.31		
	**Girls**	9.75	0.85	2.15		
**Willem’s method**	**All**	9.30	0.36	2.05	4.50	16.03
	**Boys**	9.38	0.43	2.10		
	**Girls**	9.21	0.31	2.06		
**Cameriere’s method**	**All**	8.74	-0.18	1.95	4.05	16.90
	**Boys**	8.68	-0.27	1.87		
	**Girls**	8.79	-0.11	1.91		
**Smith’s method**	**All**	8.99	0.06	2.05	4.31	15.60
	**Boys**	9.07	0.12	2.01		
	**Girls**	8.91	0.00	2.05		
Values are mean ± SD.					^*^ Difference was not significant.				

Paired t-test showed the mean chronological age to have significant differences with the mean ages calculated through Demirjian’s, Willem’s, and Cameriere’s methods (P < 0.001 for all). However, the difference between the mean chronological age and the mean age determined by Smith’s method was not significant (P = 0.079). In addition, the mean ages suggested by the four studied methods were not significantly different (Table 2). When stratified by gender, paired t-test results revealed that only the difference between the girls’ mean chronological age and the mean age estimated by Smith’s method was not significant (P = 0.900)

**Table 2:** Comparing the accuracy of Demirjian’s, Cameriere’s, Smith’s, and Willem’s methods using paired t-tests

**Age difference**	**Girls**	**Boys**	**Total**	**P**
	(n = 273)	(n = 264)	(n = 537)	(Total)
**Chronological age - Demirjian’s method**	0.85±0.98	0.90±1.01	0.87±1.00	0.000
**Chronological age - Willem’s method**	0.31±0.91	0.43±0.82	0.36±0.87	0.000
**Chronological age - Cameriere’s method**	-0.11±0.87	-0.27±0.85	-0.19±0.86	0.000
**Chronological age - Smith’s method**	0.00±0.81	0.12±0.83	0.06±0.63	0.079^*^
**Demirjian’s method - Willem’s method**	0.54±0.64	0.47±0.63	0.51±0.74	0.000
**Demirjian’s method - Cameriere’s method**	0.96±0.75	1.17±0.72	1.06±0.74	0.000
**Demirjian’s method - Smith’s method**	0.84±0.75	0.78±0.73	0.81±0.58	0.000
**Willem’s method - Cameriere’s method**	0.42±0.62	0.70±0.50	0.55±0.53	0.000
**Willem’s method - Smith’s method**	0.30±0.56	0.31±0.51	0.30±0.51	0.000
**Cameriere’s method - Smith’s method**	-0.12±0.62	-0.38±0.55	-0.25±0.60	0.000

According to Pearson’s correlation analysis, all of the four employed methods had significant positive linear correlations with each other and with chronological age (P < 0.001) (Table 3). Furthermore, both Demirjian’s and Willem’s methods had the greatest accuracy in determining the age of 6-11-year-old girls and boys. The estimations based on Cameriere’s method were most accurate in 6-12-year-old boys and 6-11-year-old girls. Finally, Smith’s method had the highest accuracy in calculating the age of 6-12-year-old children. On the other hand, while the underestimations using Cameriere’s method were greater in boys than in girls, the opposite was true about the overestimations made by all of the other three methods (Table 3).

**Table 3:** Comparing the accuracy of Demirjian’s, Cameriere’s, Smith’s, and Willem’s methods based on Pearson’s correlation analysis

		**Chronological age (mean ± SD)**	**Estimated age (mean ± SD)**	**Correlation coefficient**	**P**
**Demirjian’s method**	**Girls**	8.90±2.12	9.75±2.37	0.909	0.000
	**Boys**	8.95±1.96	9.86±2.21	0.888	0.000
	**Total**	8.93±2.04	9.80±2.29	0.900	0.000
**Willem’s method**	**Girls**	8.90±2.12	9.21±2.19	0.910	0.000
	**Boys**	8.95±1.96	9.38±1.88	0.910	0.000
	**Total**	8.93±2.04	9.30±2.05	0.909	0.000
**Cameriere’s method**	**Girls**	8.90±2.12	8.80±2.09	0.915	0.000
	**Boys**	8.95±1.96	8.69±1.79	0.900	0.000
	**Total**	8.93±2.04	8.74±1.95	0.907	0.000
**Smith’s method**	**Girls**	8.90±2.12	8.91±2.13	0.927	0.000
	**Boys**	8.95±1.96	9.08±1.97	0.909	0.000
	**Total**	8.93±2.04	8.99±2.05	0.919	0.000

## Discussion

Age determination is a major concern in medical and legal procedures. The present study compared the accuracy of four age determination methods (Demirjian’s, Cameriere’s, Smith’s, and Willem’s methods) based on panoramic radiographs of permanent teeth. Correlation tests revealed ages estimated by all the four methods to have positive linear correlations with chronological age. On the other hand, since paired t-test suggested significant differences between chronological age and those calculated by Demirjian’s, Cameriere’s, and Willem’s methods, the Smith’s method had the greatest accuracy among all evaluated techniques. The second-fourth accuracy levels belonged to Cameriere’s, Willem’s, Demirjian’s methods, respectively.

Similar to the majority of previous research^20,23-35^, we found Demirjian’s method to overestimate age by a mean value of 0.87 years. According to Demirjian et al., who developed the method using a sample of French-Canadian children, the technique is not necessarily accurate and valid for children of other ethnicities and may thus require modifications.^20^ Therefore, numerous researchers have assessed the accuracy of Demirjian’s method in estimating the age of subjects from different races. Grover et al. reported the method to overestimate girls’ and boys’ age by 0.56 and 0.66 years, respectively.^20^ Ogodescu et al. applied the method on a sample of Rumanian children and found it to overestimate girls’ age by 0.36 years and underestimate boys’ by 0.04 years.^21^ Meanwhile, Demirjian’s method has been proved to be completely inefficient in determining the age of 4-16-year-old Nigerian children and adolescents.^22^ Similar research in the Netherlands, England, and China have also suggested the inefficiency of the method in estimating children’s age.^23-26^ Generally, most studies have indicated Demirjian’s method to overestimate age by 0.02-3.06 years.^23-35^However, Ghadim et al. reported the method to underestimate the age of 3-14-year-old Kuwaiti children by 0.69 years.^36^Likewise, Sheikhi et al. found Demirjian’s method to underestimate the age of 5-16-year-old children and adolescents from Babol (Iran) by 0.04 years.^37^ Ethnic, environmental, nutritional, and socioeconomic differences along with differences in sample size and applied statistical tests seem to be responsible for such a wide range of results.^14^

Most Iranian studies in this field have also focused on the accuracy of Demirjian’s method. For instance, Sheikhi et al. found the method to overestimate the age of 5-16-year-old subjects from Rasht by 0.02 years.^38^ The difference between their findings and ours seems to be due to the differences in ethnicity, sample size, and the applied methods. On the other hand, many studies have reported results similar to ours. In Rafsanjan, Bagherian et al. estimated the age of 3.5-13.5-year-old children and reported the values to be 0.21 and 0.15 years higher than chronological age in girls and boys, respectively.^29^ In a study on 6-13-year-old children in Mashhad, Bagherpour et al. stated that despite the positive correlation between chronological age and the value determined by Demirjian’s method, the values were significantly different. They reported the ages of girls and boys to have been overestimated by 0.25 and 0.34 years, respectively.^28^ Likewise, Javadinejad et al. suggested the method to overestimate girls’ and boys’ age by 0.47 and 0.94 years, respectively.^27^

In the present study, Willem’s method overestimated children’s age by 0.36 years (0.30 years in girls and 0.42 years in boys). Consistent results have also been indicated by previous research. Galić et al. found Willem’s method to overestimate girls’ and boys’ age by 0.24 and 0.42 years, respectively.^39^ Grover et al. reported the method to estimate girls’ and boys’ age 0.24 and 0.36 years higher than their chronological age, respectively.^20^ According to Nik-Hussein et al., ages determined by Willem’s method were overestimated by 0.10 and 0.20 years in girls and boys, respectively.^40^ Similarly, Balwant et al. revealed the method to overestimate age by about 0.24 years.^41^ In a study on 3-18-year-old children and adolescents in Isfahan (Iran), Javadinejad et al. reported that girls’ and boys’ ages determined by Willem’s method were respectively 0.06 and 0.22 years higher than their chronological age.^27^ These findings suggest that the difference between chronological age and the values determined by Willem’s method are less ethnicity-dependent (compared to Demirjian’s method) and the method can be employed with acceptable accuracy in most cases.

Various studies have been performed on the accuracy of Cameriere’s method. Cameriere et al. tested their formula on a large sample of European children and found it to underestimate age by 0.11 years.^42^ The method also underestimated age by 0.18 years (0.11 years in girls and 0.27 years in boys) in the current study and the second highest accuracy after Smith’s method. De Luca et al. concluded that Cameriere’s method could accurately estimate the age of Mexican children.^43^Likewise, Javadinejad et al. found the method to be highly accurate in determining the age of 5-15-year-old children in Isfahan (Iran).^44^ In contrast, Galić et al. reported Cameriere’s method to overestimate age by 0.09 years in boys and 0.02 years in girls.^39^ In a study to assess the accuracy of Cameriere’s method among Brazilian children, Fernandes et al. found the method to overestimate age in 5-10-year-olds and to underestimate age in 5-11-year-olds.^45^ Balwant et al. applied Cameriere’s method to determine the age of 273 children in Haryana (India). Since the method overestimated age by 0.60 years in girls and 0.70 years in boys, the authors concluded that the formula proposed by Cameriere et al. lacked adequate accuracy in age determination among Indian children. They thus highlighted the need for a new formula to estimate age in this ethnic group.^41^ The relative inconsistency between the findings of these studies and ours can be justified by differences in sample size, ethnicity, environmental characteristics, and applied statistical methods.

In the current research, Smith’s method, the modified version of the method developed by Moorrees, Fanning, and Hunt,^18^ overestimated children’s age by 0.06 years (0.12 years in boys and < 0.01 years in girls). Unfortunately, few studies have examined the accuracy of this method. In Colombia, Corral et al. compared six age determination techniques based on panoramic radiographs and found those suggested by Moorrees et al. and Smith to have the least tendency to overestimate age. Similar to our finding, they indicated these two methods as the most accurate among the six methods.^46^ In a study to compare the accuracy of Demirjian’s method and the technique developed by Moorrees et al. in estimating the age of 3-16-year-old South African subjects, Philips et al. reported the first method to most probably overestimate age while the latter tended to underestimate age. However, in contrast to our findings, they reported Demirjian’s method to have higher accuracy.^47^ Such an inconsistency could have been caused by the use of Smith’s method (instead of Moorrees et al.’s) in our study, the considerable ethnic difference between Iranian and African children, and wider age range in Philips et al.’s study. Meanwhile, according to our findings, Smith’s method was highly accurate in determining age in both genders (especially girls). Therefore, considering its simple application, it can be employed in orthodontic treatment planning or legal medicine to estimate children and adolescents’ age. Nevertheless, further research to evaluate the accuracy and validity of this method is still warranted.

Limited studies have compared various age determination methods. In London, Liversidge et al. conducted a large study to compare 15 age determination methods based on radiographs of developing teeth and reported Willem’s method to have the highest accuracy.^24^ In Egypt, El-Bakary et al. compared Willem’s and Cameriere’s methods and found that although the values determined by both methods had significant correlations with chronological age, Willem’s method had greater accuracy.^48^ Conversely (and probably due to ethnic differences), our study revealed Cameriere’s method to have higher accuracy. In a study on Bosnian-Herzegovian children, Galić et al. compared Haavikko’s, Willem’s, and Cameriere’s methods and reported them in terms of accuracy Cameriere’s and Willem’s methods to be the most and least accurate, respectively.^39^ We also found Cameriere’s method to have higher accuracy than Willem’s method. Similarly, other studies to compare Cameriere’s and Willem’s methods indicated the latter to show higher accuracy.^20,41,49^

The current study demonstrated Demirjian’s method to have the highest accuracy in 6-10-year-olds of both genders. In Rumania, Ogodescu et al. reported the highest accuracy of Demirjian’s method in 5.5-13-year-old boys and 6.5-11.5-year-old girls. They also found the method to underestimate boys’ age and overestimate girls’ age.^21^ However, many studies have reported contrasting results^23-35^ which might be attributed to differences in ethnicity, sample size, and statistical analyses. Hegde et al. suggested Demirjian’s method to show the highest accuracy in 6-12-year-old boys and girls.^50^Similar to our findings, Corral et al. suggested Demirjian’s and Smith’s methods to have the highest accuracy among 5.5-12 and 6-12 year-old children, respectively.^46^ According to Liversidge et al., most radiographic age determination methods in 3-15-year-old children tend to overestimate younger ages and underestimate older ages.^50^

Since the studied methods had distinct tables and indices for the two genders, their accuracy needs to be evaluated separately in each gender. Based on our findings, all four methods had lower error rates in girls than in boys. However, this difference was not statistically significant. Besides, only girls’ age determined by Smith’s method did not have a significant difference with their chronological age. Other studies have also mentioned lower error rate of radiographic age determination methods in girls, but have failed to establish significant differences between the rates in two genders.^22,23,40,43,45^

## Conclusion

The present research showed that although Smith’s method had the highest accuracy in estimating the age of the studied sample, Cameriere’s, Willem’s, and Demirjian’s methods can still determine Iranian children and adolescents’ age with acceptable accuracy.
